# Diseases of the Circulation

**Published:** 1906-04-28

**Authors:** 


### DISEASES OF THE CIRCULATION.

**Manipulation of the heart** in case of sudden failure from shock has been shown to be of practicable value. W. S. Conkling [1] reports the case of a man suffering from a large wound of the chest wall, where the pulse had ceased apparently for two minutes. Introducing his finger and thumb into a rent of the pericardium, he felt the motionless heart and manipulated it for nearly a minute till a movement took place. After stimulants, and saline injections the patient recovered. Manipulation through the diaphragm has been recorded in three successful cases and is likely to be of use during abdominal operations when the heart fails. H. M. Gray [2] made an incision in the middle line below the xiphoid cartilage with a penknife in an urgent case. He then used intermittent pressure with two fingers about seventy times a minute; at the end of four minutes the heart became hard, and then a tremor was felt. After this it began to beat slowly and gradually increased its force and rate. Artificial respiration had been kept up, but the patient soon began to breathe normally, and the colour returned to the face. It was thought that about nine minutes elapsed from the time when the heart stopped to that when it again began to beat. Crile also mentions an instance when the heart stopped for nine minutes and was recovered by massage, adrenalin, and artificial respiration.

*Congenital Malformations* of the heart are generally due to a germinal defect rather than to antenatal disease, according to Bosanquet.[3] For they are apt to appear in several members of a family and to be accompanied by other malformations. The cause of cyanosis is uncertain, but it is not due to admixture of the blood from the two sides of the heart, nor to failure of the circulation. A more probable cause is the great concentration of the blood and its richness in corpuscles and hæmoglobin, which is thought to be an attempt at compensation. The diagnosis of the various forms of cardiac lesions is difficult and uncertain. A systolic murmur in the pulmonary area, with a thrill, cyanosis, and clubbed fingers, is suggestive of stenosis of the pulmonary artery. A systolic murmur alone is often due to to a patent foramen ovale or ductus arteriosus, though these lesions may give murmurs diastolic in time. Accentuation of the second sound in the pulmonary area is caused by one or other of these same lesions. Stenosis of the aorta is shown by a systolic murmur to the right of the sternum, and insufficiency of the tricuspid valve by a systolic murmur lower down with venous pulsation and cyanosis.

**Functional Murmurs.**—J. E. Squire [4] points out that hæmic murmurs are generally at the base. Those confined to the apex are really due to regurgitation, though of a temporary character. A systolic murmur in the pulmonary area, if not hæmic, may be due to (1) congenital stenosis, (2) to displacement of the heart and bending of the pulmonary artery as in various pulmonary conditions, (3) pressure of a tumour, (4) or retraction of the covering lung. A murmur heard at the junction of the seventh left cartilage with the sternum is oftentimes due to pericardial thickening, while the sounds of pleural friction may have a to and fro character due to movements communicated from the heart. Another pseudo-murmur of presystolic character is sometimes heard when an adherent pericardium exists. In life assurance a question may arise whether a murmur is organic, or cardio-respiratory. An important type of the latter is a whiffing, high-pitched sound during the systole of the heart, produced by the kick of the ventricle or aorta pressing out the air from the overlying tongue of lung-tissue. It is generally audible only during part of the respiratory cycle, usually during inspiration alone. It may easily be mistaken for a true murmur at the apex, but if heard just below, and inside the tip of the left scapula, a mistake can hardly be made. The writer does not think this murmur is a sign of tuberculosis, as has been asserted, or, in short, of any importance, and at one time he had such a murmur in his own chest. In cases of doubt it is well to remember that these pseudo-murmurs frequently disappear on holding the breath, and are louder or entirely disappear during one part of the respiratory cycle. It is useful in case of doubt to auscultate over the trachea where these cardio respiratory murmurs may be heard, or to make the patient lie down, which will often make them vanish. Finally, the normal heart sounds sometimes give an echo if there are cavities in a diseased lung which curiously simulates a murmur.

*Aneurysm of the Abdominal Aorta.*—Osler [5] has written a most instructive monograph on this somewhat rare condition, which, as he says, is very often diagnosed when not present, and when present may be easily overlooked and the symptoms put down to other diseases. He found 16 cases among 18,000 in-patients at the Johns Hopkins Hospital, while his thoracic aneurysms were ten times as numerous. It is much rarer in women than in men, though a throbbing aorta is so common in them. The majority of patients are under forty, and have suffered from syphilis. The pain begins early and remains throughout of frightful intensity. It may be due to pressure on the nerves, erosion of the vertebræ, or to rupture of the sac and diffusion of blood among the tissues. The dull boring pain alternates with dreadful paroxysms. Vomiting, constipation, and pressure symptoms may occur in some cases, as well as visible pulsation. On the other hand, in many healthy persons this pulsation can be seen, often in two spots, one just below the ensiform cartilage and the other just above and to the left of the navel. When the right side of the heart is enlarged, pulsation in the upper part of this area is often marked either from the impulse given to the left lobe of the liver or from the direct protrusion of the ventricle. In enteroptosis and emaciation, the aorta itself may become noticeable. In neurotic women abnormal pulsation is, of course, common, and may be accompanied with small hæmorrhages from the stomach and bowels. However, no pulsation, thrill, or bruit, without the presence of a palpable expansile tumour should be taken to indicate an abdominal aneurysm. Tumours overlying the vessels may pulsate strongly, but are not expansile. In anæmia there may be a bounding impulse with a thrill and bruit of marked character, and the same thing may occur when the aortic valves are insufficient. A sclerotic aorta in an emaciated subject, again, may suggest aneurysm. Osler thinks the most serious difficulty is met with when an aneurysm has ruptured and formed a large tumour with little or no pulsation. This has been often opened in mistake with disastrous results, especially as the anæmia, wasting, and slight fever cause great similarity to the effects of a new growth. Occasionally a cure may take place naturally or as the result of treatment. Rest, with low diet or gelatine injections may be tried. Ligation of the aorta has always been fatal, but compression above the sac, or the introduction of a coil of wire through which an electric current is passed has given slightly better results. The extraordinary way in which life may be carried on for some time after the blood has made its way into the surrounding tissues is shown in a case of dissecting aneurysm of the aorta recently reported by Letulle.[6] The blood had forced its way between the coats of the aorta and formed a new channel surrounded by a thick layer of connective tissue. It entered this channel just above the aortic valves and returned to the abdominal aorta at its bifurcation by a second opening, thus forming a secondary aorta through which the blood circulated. Death took place nearly three months after the first symptoms were noticed.

**Tricuspid Stenosis.**—D. Halliday Croom[7] describes a case where the associated pulsation in the liver was ventricular in character instead of being of an auricular type which is usually the case. The patient also suffered from tricuspid incompetence and mitral stenosis. The latter frequently accompanies the tricuspid lesion. Double thrills were felt both to the right and to the left of the sternum, and the dulness was extended four or five inches on both sides of the midsternal line. Besides murmurs in other situations there were presystolic and systolic ones near the right nipple. Cyanosis was marked and when the blood was counted the red cells amounted to 8,800,000. Under treatment by rest and digitalis this number was reduced, but it was found that oxygen inhalations had an even greater effect in reducing the cyanosis and excess of red blood-cells. The diagnosis was confirmed by post-mortem findings. A similar case in some respects is reported by Bond Shaw[8] who gives reasons for thinking that the tricuspid stenosis was here congenital, though it is remarkable that it caused no disturbance for 28 years till the mitral valve failed. It is noticeable, too, that no murmur was heard over the tricuspid area, though cyanosis and pulsation of the liver were present.

*Echinococcus.*—Bland Sutton[9] shows that when the *T. echinococcus* invades the heart, the colonies nearly always appear in the subserous areolar tissue, as indeed they do in other organs. This tissue in the heart is found notably in the auriculo-ventricular groove, and here, in most instances, the parasite lodges. Cysts may exist without symptoms for long periods and then cause sudden death. In other cases they may cause symptoms of valve lesions, or they may burst into the heart and lead to a shower of emboli producing gangrene or sudden paralysis or death.

[1] Lancet, Sept. 30. [2] Lancet, Aug. 19. [3] Clinical Jour., Jan. 24. [4] Clinical Jour., Sept. 13. [5] Lancet, Oct. 14. [6] Lancet, Feb. 3. [7] Edin. Med. Jour., Sept. [8] Amer. Jour. Med. Sc., Aug. [9] Clinical Jour., Nov. 22.

